# Mechanisms of Aquaporin-Facilitated Cancer Invasion and Metastasis

**DOI:** 10.3389/fchem.2018.00135

**Published:** 2018-04-25

**Authors:** Michael L. De Ieso, Andrea J. Yool

**Affiliations:** Department of Physiology, Adelaide Medical School, University of Adelaide, Adelaide, SA, Australia

**Keywords:** aquaporin, cell migration, metastasis, cancer, invasion, pharmacology, drug

## Abstract

Cancer is a leading cause of death worldwide, and its incidence is rising with numbers expected to increase 70% in the next two decades. The fact that current mainline treatments for cancer patients are accompanied by debilitating side effects prompts a growing demand for new therapies that not only inhibit growth and proliferation of cancer cells, but also control invasion and metastasis. One class of targets gaining international attention is the aquaporins, a family of membrane-spanning water channels with diverse physiological functions and extensive tissue-specific distributions in humans. Aquaporins−1,−2,−3,−4,−5,−8, and−9 have been linked to roles in cancer invasion, and metastasis, but their mechanisms of action remain to be fully defined. Aquaporins are implicated in the metastatic cascade in processes of angiogenesis, cellular dissociation, migration, and invasion. Cancer invasion and metastasis are proposed to be potentiated by aquaporins in boosting tumor angiogenesis, enhancing cell volume regulation, regulating cell-cell and cell-matrix adhesions, interacting with actin cytoskeleton, regulating proteases and extracellular-matrix degrading molecules, contributing to the regulation of epithelial-mesenchymal transitions, and interacting with signaling pathways enabling motility and invasion. Pharmacological modulators of aquaporin channels are being identified and tested for therapeutic potential, including compounds derived from loop diuretics, metal-containing organic compounds, plant natural products, and other small molecules. Further studies on aquaporin-dependent functions in cancer metastasis are needed to define the differential contributions of different classes of aquaporin channels to regulation of fluid balance, cell volume, small solute transport, signal transduction, their possible relevance as rate limiting steps, and potential values as therapeutic targets for invasion and metastasis.

## Introduction

### Aquaporins

Aquaporins (AQPs) are a family of water channels that also include a subset of classes shown to mediate transport of glycerol, ions, and other molecules (Li and Wang, 2017). The first aquaporin to be cloned, aquaporin-1 (AQP1), was identified in red blood cells and renal proximal tubules (Denker et al., 1988; Preston and Agre, 1991). In the *Xenopus laevis* expression system, introduced AQP1 channels enabled high osmotic water flux across the plasma membrane as compared to non-AQP control oocytes (Preston et al., 1992), explaining the mechanism enabling rapid transmembrane passage of water in certain types of cells. To date, 15 classes of aquaporin genes have been identified in mammals (AQP0–AQP14), with AQPs 13 and 14 found in older lineages of mammals (Metatheria and Prototheria) (Ishibashi et al., 2009; Finn et al., 2014; Finn and Cerda, 2015). The first 13 aquaporins (AQP0–AQP12) have been divided into categories based on functional properties (Li and Wang, 2017). One comprises the classical aquaporins (AQP0,−1,−2,−4,−5,−6,−8), which were thought initially to transport only water, though some also transport gases, urea, hydrogen peroxide, ammonia, and charged particles (Ehring and Hall, 1988; Preston et al., 1992; Fushimi et al., 1993; Hasegawa et al., 1994; Raina et al., 1995; Ma et al., 1996, 1997a; Chandy et al., 1997; Ishibashi et al., 1997b; Yasui et al., 1999; Anthony et al., 2000; Nakhoul et al., 2001; Bienert et al., 2007; Herrera and Garvin, 2011; Almasalmeh et al., 2014; Rodrigues et al., 2016). A second category consists of the aquaglyceroporins (AQP3,−7,−9, and−10), which are permeable to water and glycerol, with some also exhibiting urea, arsenite, and hydrogen peroxide permeability (Ishibashi et al., 1997a, 1998, 2002; Yang and Verkman, 1997; Liu et al., 2002; Lee et al., 2006; Rojek et al., 2008; Miller et al., 2010; Watanabe et al., 2016). A possible third category consists of AQP11 and AQP12, distantly related paralogs with only 20% homology with other mammalian AQPs (Ishibashi, 2009), which appear to carry both water and glycerol (Yakata et al., 2011; Bjørkskov et al., 2017). The permeability of AQP11 to glycerol could be important for its function in human adipocytes, in which it is natively expressed (Madeira et al., 2014). Aquaporins assemble as homo-tetramers, with monomers ranging 26–34 kDa (Verkman and Mitra, 2000). In most AQPs, each monomer is composed of six transmembrane domains and intracellular amino and carboxyl termini, with highly conserved asparagine-proline-alanine (NPA) motifs in cytoplasmic loop B and in extracellular loop E (Jung et al., 1994). The NPA motifs in loops B and E contribute to a monomeric pore structure that mediates selective, bidirectional, single-file transport of water in the classical aquaporins (Sui et al., 2001), and water and glycerol in aquaglyceroporins (Jensen et al., 2001).

Intracellular signaling processes regulate AQP channels by altering functional activity, intracellular localization, and levels of expression in different cells and tissues. For example, the peptide hormone vasopressin regulates excretion of water in the kidney by augmenting water permeability of collecting duct cells. Vasopressin induces phosphorylation of AQP2 (Hoffert et al., 2006), stimulating the reversible translocation of AQP2 from intracellular vesicles to the apical plasma membrane (Nielsen et al., 1995). Guanosine triphosphate (GTP) stimulates AQP1-induced swelling of secretory vesicles in the exocrine pancreas (Cho et al., 2002), with functional implications in pancreatic exocrine secretions. Additionally, AQP1 ion channel activity is activated by intracellular cGMP (Anthony et al., 2000), and phosphorylation of Y253 in the carboxyl terminal domain regulates responsiveness of AQP1 ion channels to cGMP (Campbell et al., 2012). Given the diverse array of functional properties, mechanisms of regulation, and tissue-specific distributions being discovered for aquaporins, it is not surprising that different classes of aquaporins (AQP-1,−2,−3,−4,−5,−8, and−9) have been implicated specifically in the complex steps associated with cancer invasion and metastasis (Table 1), suggesting specialized roles for these channels have been arrogated into the pathological processes.

**Table 1:** Key roles of AQPs involved in cancer invasion and metastasis.

**AQP**	**Permeable to:**	**Key physiological role(s)**	**Cancer(s) up-regulated**	**Key role(s) in cancer invasion and metastasis**
AQP1	•Water (Preston et al., 1992), monovalent cations (Anthony et al., 2000), CO_2_ (Nakhoul et al., 1998), H_2_O_2_ (Almasalmeh et al., 2014), NO (Herrera et al., 2006), and NH_3_ (Nakhoul et al., 2001)	•Water reabsorption in proximal tubule of the kidney for concentrating urine (Ma et al., 1998; Schnermann et al., 1998) •Secretion of aqueous fluid from ciliary epithelium in the eye, and cerebrospinal fluid from the choroid plexus (Zhang et al., 2002; Oshio et al., 2005) •Perception of thermal inflammatory pain and cold-induced pain (Zhang and Verkman, 2010)	Glioma (Saadoun et al., 2002a; El Hindy et al., 2013), mammary carcinoma (Endo et al., 1999), lung adenocarcinoma (Hoque et al., 2006), colorectal carcinoma (Moon et al., 2003), hemangioblastoma (Chen et al., 2006), and multiple myeloma (microvessels) (Vacca et al., 2001)	•Upregulated in response to tumor tissue hypoxia. Enables recruitment of new tumor vasculature by enhancing endothelial cell migration •Polarizes to leading and trailing edge of migrating cell, and enhances tumor cell migration and invasion by enabling rapid membrane protrusion formation via cell volume regulation and interaction with cytoskeletal dynamics •Enhances mesenchymal stem cell migration via FAK and β-catenin pathways •Might contribute to EMT •Possible interaction with ECM-degrading proteases
AQP2	•Water (Fushimi et al., 1993)	•Water reabsorption in collecting duct of the kidney to concentrate urine (Rojek et al., 2006)	Endometrial carcinoma (Zou et al., 2011)	•Enables “traction” for migrating cell by contributing to the regulation and recycling of focal adhesion proteins (e.g., integrin) •Necessary in estradiol-induced invasion and adhesion of endometrial carcinoma cells, through reorganization of F-actin
AQP3	•Water (Echevarria et al., 1994), glycerol, urea (Ishibashi et al., 1994), H_2_O_2_ (Miller et al., 2010), arsenite (Lee et al., 2006), and NH_3_ (Holm et al., 2005)	•Water reabsorption in collecting duct of the kidney to concentrate urine (Ma et al., 2000) •Skin hydration (Ma et al., 2002) •Skin wound healing (Hara-Chikuma and Verkman, 2008a)	Lung cancer (Liu et al., 2007), hepatocellular carcinoma (Guo et al., 2013), gastric cancer (Shen et al., 2010), prostate cancer (Hwang et al., 2012), oesophageal and oral squamous cell carcinoma (Kusayama et al., 2011), colorectal carcinoma (Moon et al., 2003), skin squamous cell carcinoma (Hara-Chikuma and Verkman, 2008b), ovarian cancer (Ji et al., 2008), pancreatic cancer (Direito et al., 2017), and breast cancer (Mobasheri and Barrett-Jolley, 2014)	•Upregulated by EGF, and contributes to EGF-induced EMT and cancer migration •Contributes to chemokine-dependent cancer migration via enabling H_2_O_2_ influx and its downstream cell signaling •Interacts with ECM-degrading proteases •Might enhance tumor cell migration and invasion via regulation of cell protrusion formation
AQP4	•Water (Hasegawa et al., 1994)	•Water reabsorption in collecting duct of the kidney to concentrate urine (Ma et al., 1997b) •Transport of water into and out of the brain and spinal cord via blood-brain barrier (Manley et al., 2000) •Neuroexcitation (Binder et al., 2006) •Enables astrocyte cell migration following injury (Saadoun et al., 2005b)	Glioma (Saadoun et al., 2002b) and meningioma (Ng et al., 2009)	•Co-localizes with ion channels at leading and trailing edges of migrating cancer cells •Enhances tumor cell migration and invasion by enabling rapid membrane protrusion formation via cell volume regulation and interaction with cytoskeletal dynamics •Might interact with ECM-degrading proteases
AQP5	•Water (Raina et al., 1995) and H_2_O_2_ (Rodrigues et al., 2016)	•Secretion of saliva (Ma et al., 1999) and airway mucus (Song and Verkman, 2001)	Prostate cancer (Li et al., 2014), chronic myelogenous leukemia (Chae et al., 2008a), colorectal carcinoma (Wang et al., 2012), hepatocellular carcinoma (Guo et al., 2013), lung cancer (Chae et al., 2008b), cervical cancer (Zhang et al., 2012), pancreatic cancer (Direito et al., 2017), and breast cancer (Jung et al., 2011)	•Promotes EMT •Co-localizes with ion channels at leading and trailing edges of migrating cancer cells •Enhances tumor cell migration and invasion by enabling rapid membrane protrusion formation via cell volume regulation •Might interact with EGFR/ERK1/2 signaling pathway
AQP8	•Water, urea (Ma et al., 1997a), H_2_O_2_ (Bienert et al., 2007), and NH_3_ (Holm et al., 2005; Saparov et al., 2007)	•Canalicular bile water secretion (Calamita et al., 2005) •Colonic water reabsorption (Yamamoto et al., 2007)	Cervical cancer (Shi et al., 2012, 2014)	•Not yet known
AQP9	•Water, urea (Ishibashi et al., 1998), glycerol (Tsukaguchi et al., 1998), arsenite (Liu et al., 2002), and H_2_O_2_ (Watanabe et al., 2016)	•Hepatic glycerol uptake and metabolism for glucose production (Kuriyama et al., 2002; Rojek et al., 2007; Maeda et al., 2009) •Route for excretion of arsenic by the liver (Carbrey et al., 2009) and modulates arsenic sensitivity in leukemia (Bhattacharjee et al., 2004; Leung et al., 2007)	Glioblastoma (Fossdal et al., 2012), astrocytoma (Tan et al., 2008), prostate cancer (Chen et al., 2016)	•Overexpression might correspond with reduced EMT and growth in hepatocellular carcinoma •Might interact with ERK1/2 and MMP9 to enhance prostate cancer invasion and migration

### Cancer invasion and metastasis

**Figure 1:**
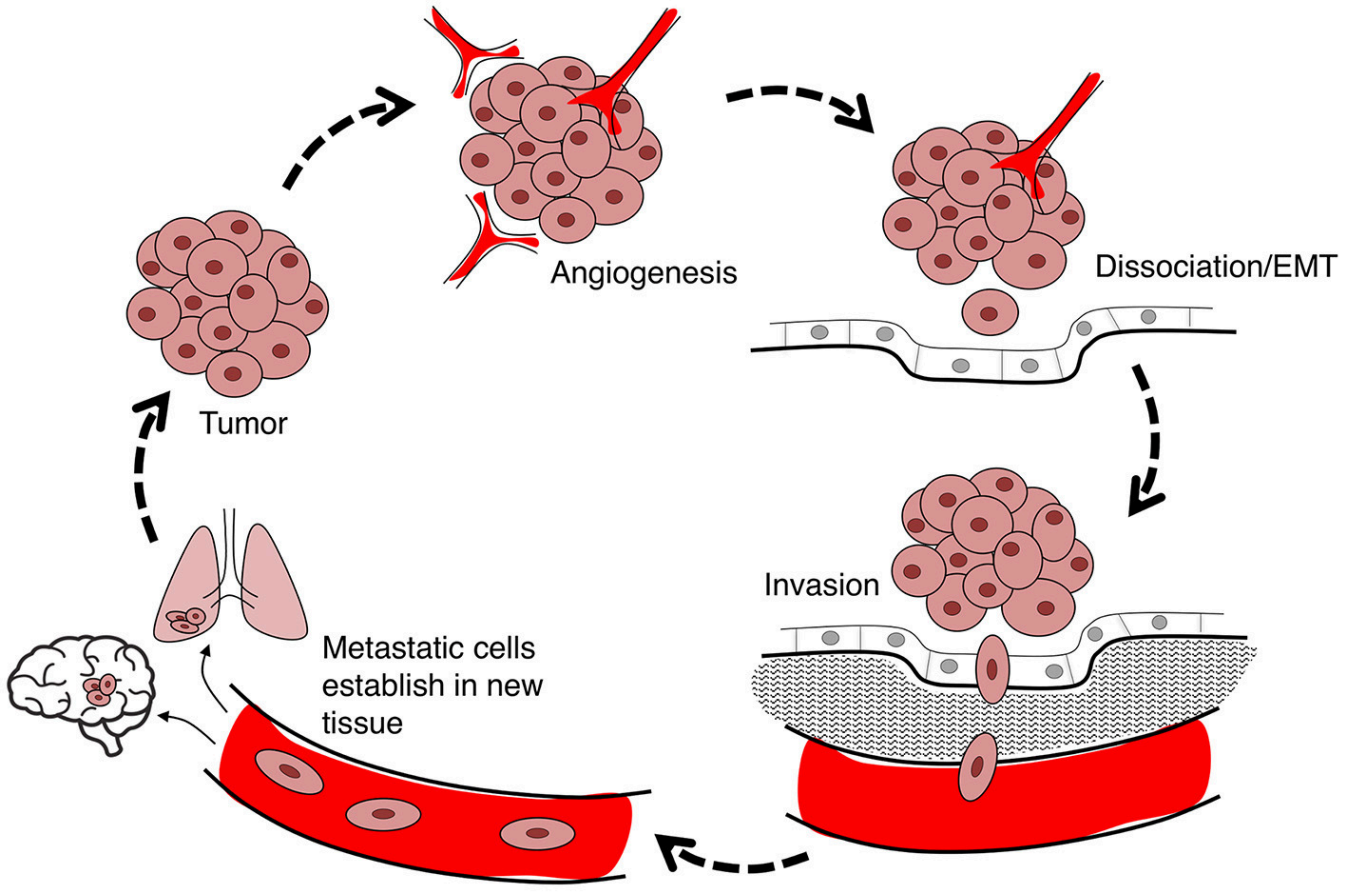
Flow diagram summarizing the steps in cancer metastasis. Metastasis involves the migration of cells from the primary tumor to distant organs. Large tumors with tissue hypoxia rely on angiogenesis for vascular exchange of nutrients and waste. Primary tumor cells undergo phenotypic changes including loss of cell-cell adhesions which enables cells to dissociate from primary tumor, invade the adjacent extracellular matrix (ECM), and intravasate into the blood or lymph systems. Circulating tumor cells extravasate to seed secondary sites at which the process can reoccur.

Cancer is a leading cause of death worldwide, accounting for 8.2 million deaths in 2012 (Ferlay et al., 2015). The incidence of cancer is rising steadily in an aging population, with numbers expected to increase 70% in the next two decades (Ferlay et al., 2015). Current treatments involve chemotherapy, radiation therapy, and surgery (Miller et al., 2016), associated with an array of side effects including nausea (Koeller et al., 2002), impaired fertility and premature menopause (Howard-Anderson et al., 2012; Wasilewski-Masker et al., 2014), painful neuropathy (Gamelin et al., 2002; Rivera and Cianfrocca, 2015), increased risk of cardiovascular disease (Monsuez et al., 2010; Willemse et al., 2013), and loss of bone density (Gralow et al., 2013). Inhibiting proliferation remains the primary focus of cancer treatments, although the predominant cause of death is cancer metastasis (Yamaguchi et al., 2005; Spano et al., 2012). Less devastating cancer therapies might be achievable via a combination of strategies that not only inhibit proliferation, but also control metastasis of tumor cells from their primary site to distant organs (Friedl and Wolf, 2003). Cancer cell migration through the body exploits pathways including blood stream, lymphatic system, and transcoelomic movement across body cavities (Wyckoff et al., 2000; Pepper et al., 2003; Tan et al., 2006). The hierarchical nature of the metastatic cascade suggests it should be vulnerable to intervention at multiple levels including angiogenesis, detachment of cells from the primary tumor, and infiltration of dissociated tumor cells into and out of circulatory pathways via intravasation and extravasation, respectively (Figure 1). AQPs that serve as rate-limiting steps in the metastatic cascade should have substantial value as prognostic markers and pharmacological targets for treatments.

## Angiogenesis

Both cancer invasion and metastasis are enhanced by angiogenesis. Angiogenesis, activated in response to inadequate oxygen perfusion, triggers extracellular matrix breakdown; endothelial cell proliferation, differentiation, and migration; and recruitment of periendothelial cells (Clapp and de la Escalera, 2006) which form discontinuous layers around vessels and exert developmental and homeostatic control (Njauw et al., 2008). Under physiological conditions, angiogenesis is seen in the proliferative phase of the menstrual cycle (Demir et al., 2010), development of fetal and placental vasculature (Demir et al., 2007), and skeletal muscle following physical activity (Egginton, 2009). In pathological scenarios such as tumorigenesis, tissue hypoxia stimulates the formation of new vasculature, enabling tumors to better obtain nutrients, exchange gases, and excrete waste (Nishida et al., 2006). Folkman et al. (1966) showed that tumors up to 2 mm in diameter could survive via passive diffusion from surrounding tissue; but angiogenesis was essential for support of larger tumors.

AQP1, expressed in peripheral vascular endothelial cells, is involved in tumor angiogenesis (Nielsen et al., 1993; Endo et al., 1999; Saadoun et al., 2002a; El Hindy et al., 2013; Verkman et al., 2014). AQP1 knock-down in chick embryo chorioallantoic membrane resulted in a dramatic inhibition of angiogenesis (Camerino et al., 2006). Saadoun et al. (2005a) found AQP1-deficient mice exhibited reduced tumor growth and angiogenesis as compared to wild type, following subcutaneous or intracranial B16F10 melanoma cell implantation. Their work showed AQP1-null endothelial cells from mouse aorta had reduced motility as compared to wild-type, suggesting AQP1 was needed to facilitate cell migration for angiogenesis. Monzani et al. (2009) confirmed a reduced migration capacity in human microvascular endothelial cells (HMEC-1) after AQP1 knockdown by siRNA. AQP1 mRNA and protein levels are increased in response to tissue hypoxia (Kaneko et al., 2008; Abreu-Rodríguez et al., 2011). AQP1 facilitates hypoxia-induced angiogenesis by enhancing endothelial cell migration.

Angiogenesis is regulated by growth factors such as vascular endothelial growth factor (VEGF), which stimulates endothelial cell proliferation and angiogenesis in response to hypoxia (Suzuki et al., 2006), through processes that could augment AQP1 activity indirectly. Pan et al. (2008) found a positive correlation between levels of AQP1 expression, intratumoral microvascular density, and VEGF in endometrial adenocarcinoma. Similarly, AQP1 gene deletion correlated with reduced VEGF receptor expression in mouse primary breast tumor cells (Esteva-Font et al., 2014), and knockdown of AQP1 in human retinal vascular endothelial cells with concurrent inhibition of VEGF caused an additive inhibition of hypoxia-induced angiogenesis (Kaneko et al., 2008). However, application of VEGF-neutralizing antibodies did not alter AQP1 expression (Kaneko et al., 2008), and levels of VEGF in primary breast tumors were not different between AQP1-null and wild-type mice (Esteva-Font et al., 2014), supporting the idea that VEGF is regulated independently of AQP1 expression or activity.

Other angiogenic factors, such as hypoxia-inducible factor 1-alpha (HIF-1α), induce AQP1 expression in low oxygen conditions (Abreu-Rodríguez et al., 2011). The AQP1 gene promoter carries a HIF-1α binding site which drives AQP1 expression in response to hypoxia in cultured human retinal vascular endothelial cells (HRVECs) (Tanaka et al., 2011), and involves phosphorylation of p38 mitogen-activated protein kinase (MAPK) (Tie et al., 2012). Estrogen signaling also targets the promoter region of the AQP1 gene to increase transcription, inducing enhanced tubulogenesis of vascular endothelial cells as a model for angiogenesis (Zou et al., 2013). In summary, AQP1 is upregulated by angiogenic factors in response to hypoxia, and necessary for endothelial cell migration and angiogenesis. Therapies aimed at blocking transcriptional activation of AQP1 could impede cancer angiogenesis, if the treatment could be spatially limited to the tumor site without impacting normal cell functions.

## Cellular dissociation and epithelial-mesenchymal transition

Epithelial-mesenchymal transition (EMT) occurs in normal physiological conditions such as implantation, embryogenesis, and organ development, as well as pathological processes such as cancer invasion and metastasis (Vićovac and Aplin, 1996; Thiery, 2002). During EMT, polarized epithelial cells undergo biochemical changes to adopt a mesenchymal phenotype, characterized by a loss of cell polarity, reduced cell-cell adhesiveness, and enhanced invasive capacity (Thiery, 2002, 2003; Cavallaro and Christofori, 2004; Kalluri and Weinberg, 2009; van Zijl et al., 2011). Epithelial cadherin (E-cadherin), a transmembrane glycoprotein, enables calcium-dependent tight adhesions between epithelial cells and links to cytoskeletal elements (Angst et al., 2001; Alizadeh et al., 2014). Downregulation of E-cadherin is a hallmark feature of EMT (Cano et al., 2000; Chua et al., 2007; Korpal et al., 2008). EMT in cancer is induced by signals from the tumor-associated stroma, including epidermal growth factor (EGF), platelet-derived growth factor (PDGF), hepatocyte-derived growth factor (HGF), and transforming growth factor beta (TGF-β) (Miettinen et al., 1994; Pagan et al., 1999; Lo et al., 2007; Kong et al., 2009; Xu et al., 2009). These signals stimulate transcription factors such as SNAI1 (SNAIL), SNAI2 (SLUG), zinc finger E-box binding homeobox 1 (ZEB1), Mothers against decapentaplegic homolog 2 (SMAD-2) and Twist, which are all E-cadherin transcription repressors (Yang et al., 2004; Medici et al., 2008).

Classes of aquaporins such as AQP3 have been implicated in the EMT process. AQP3 up-regulation in response to EGF in colorectal, gastric, and pancreatic cancers, is associated with augmented cell migration, invasion, and metastasis (Huang et al., 2010; Liu et al., 2012; Li et al., 2013). In gastric cancer, EGF-induced AQP3 upregulation enhances the mesenchymal transformation (Chen et al., 2014). Chen et al. (2014) determined that mRNA and protein levels of vimentin and fibronectin (proteins associated with mesenchymal phenotype) were significantly increased in cells with high levels of AQP3 expression but decreased in AQP3-deficient cells. Conversely, E-cadherin expression was significantly lower in cells with high AQP3 and increased in AQP3-knockdown cells. The mechanisms for AQP3-facilitated pancreatic and colorectal cancer cell migration have not yet been determined. It will be interesting to investigate whether AQP3 promotes EMT in these cancers.

In addition to AQP3, AQPs 1, 4, 5, and 9 also have been linked to EMT in different types of cancer cells. In lung adenocarcinoma cells, AQP1 overexpression correlated with the down-regulation of E-cadherin, and up-regulation of vimentin (Yun et al., 2016). AQP4 knockdown in human breast cancer was associated with increased levels of E-cadherin, and in glioma cells with increased β-catenin (involved in actin reorganization and cell-cell adhesion) and connexin-43 (a gap junction protein that contributes to cell-cell signaling and adhesion) (Ding et al., 2011; Li Y. et al., 2016), suggesting AQP4 might enhance cell detachment from primary tumors. However, opposing evidence showed knockdown of AQP4 in primary human astrocytes correlated with down-regulation of connexin-43 (Nicchia et al., 2005); and transfection of wild type AQP4 into glioma cell lines caused enhanced adhesion (McCoy and Sontheimer, 2007). In primary glial cells, AQP4 expression levels had no appreciable effect on cell-cell adhesion under the conditions tested (Zhang and Verkman, 2008). In human non-small cell lung cancer cells (NSCLCs), AQP5 increased invasiveness; conversely, expression of AQP5 mutant channels lacking membrane targeting signals or the S156 phosphorylation site did not augment invasiveness (Chae et al., 2008b). Overexpression of AQP5 in NSCLCs was associated with a reduction in epithelial cell markers such as E-cadherin, α-catenin, and γ-catenin, and an increase in mesenchymal cell markers such as fibronectin and vimentin, concomitant with a mesenchymal change in morphology. Similarly, AQP3 and AQP5 overexpression in pancreatic ductal adenocarcinoma is accompanied by downregulation of E-cadherin and upregulation of vimentin (Direito et al., 2017). The invasion-promoting properties of AQP5 expression appear to depend on the c-Src signaling pathway, a potent trigger of EMT (Guarino et al., 2007; Chae et al., 2008b). High AQP5 expression correlated with an increase in phosphorylated SMAD2, promoting EMT in colorectal cancer, whereas AQP5 silencing was associated with a down-regulation of phosphorylated SMAD2, and a repressed EMT response (Chen et al., 2017). AQP9 is downregulated in hepatocellular carcinoma; overexpression corresponds to reduced growth and EMT, thus reducing cancer invasion and metastasis (Li C. F et al., 2016; Zhang et al., 2016). Evidence suggests that AQPs have different effects depending on the type of cancer. Moreover, the state of cancer progression, environmental factors, and the types of assays used will be complicating factors; nevertheless, AQPs have clear potential as diagnostic and prognostic biomarkers, and as therapeutic targets for modulation of EMT, cell-cell adhesion, and dissociation phases of cancer progression.

## Invasion and cell migration

Cell migration involves the translocation of individual and collective groups of cells through fluid or tissues, relevant for survival in multicellular and single-celled organisms (Klausen et al., 2003; Friedl et al., 2004). Migration enables physiological morphogenesis, immunity, and tissue repair (Friedl et al., 2004; Friedl and Weigelin, 2008). In most mammalian cells, migration is highest during development and morphogenesis and decreases after terminal differentiation. In pathological circumstances such as cancer, migration machinery can be reactivated. AQPs−1,−3,−4, and−5,−8, and−9 are known to contribute to cancer cell migration and invasion. Translocation of cancer cells can be initiated by chemokines released from host tissues, and growth factors such as EGF secreted by stromal cells (Dittmar et al., 2008; Roussos et al., 2011).

AQP3 has been suggested to increase EGF-induced cancer growth and migration by mediating H_2_O_2_ flux (Miller et al., 2010; Hara-Chikuma et al., 2016). H_2_O_2_ is known as an oxidative stressor, but is also a second messenger in cell proliferation, differentiation and migration (Thannickal and Fanburg, 2000; Rhee, 2006). AQP3 knockdown in skin and lung cancer cell lines reduced EGF-induced H_2_O_2_ influx, and attenuated EGF signaling cascades (Hara-Chikuma et al., 2016), reducing migration and growth. H_2_O_2_ also influenced chemokine-dependent migration of T-cells and breast cancer cells (Hara-Chikuma et al., 2012; Satooka and Hara-Chikuma, 2016). AQP1,−3,−5,−8, and−9 have all been suggested to transport H_2_O_2_ (Bienert et al., 2007; Miller et al., 2010; Almasalmeh et al., 2014; Rodrigues et al., 2016; Watanabe et al., 2016). All of these classes also have been linked with cancer cell migration (Hu and Verkman, 2006; Shi et al., 2013; Li et al., 2014; Chen et al., 2015; Zhang et al., 2016); however, H_2_O_2_ transport has thus far been linked only to AQP3 as a control mechanism in cancer cell migration. Further work might show H_2_O_2_ transport in other classes of AQPs regulates cell motility and invasion.

### Polarization

**Figure 2:**
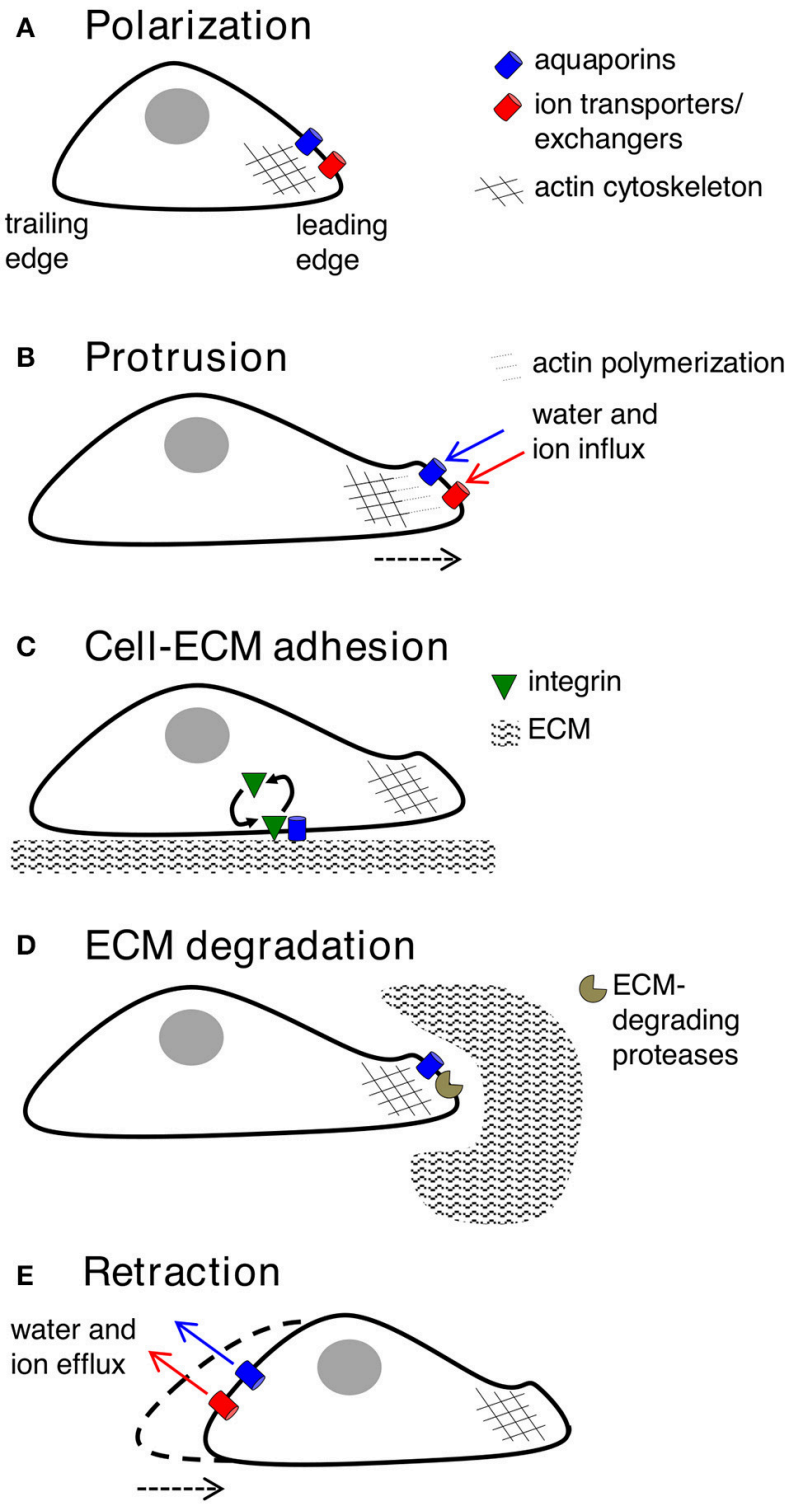
Key contributions of aquaporins in cell migration**. (A)** Forward movement is preceded by establishing specialized loci within the cell, with redistribution of aquaporins, ion transporters/exchangers, and actin polymerization machinery to the leading edge. AQP-1,−4,−5, or−9 can be found on leading edges of migrating cancer cells. **(B)** Protrusions of the membrane might use water influx (down an osmotic gradient established by ion transporters/exchangers) and actin polymerization beneath the plasma membrane to dynamically push the membrane forward. AQP-1,−4, and−5 are implicated in water influx for protrusion extension in cancer cells; AQPs-1 and−4 also appear to interact with actin cytoskeleton. **(C)** Protrusions adhere to the ECM using integrin to generate “traction” for cellular movement. AQP2 might modulate turnover of integrin at adhesion sites, enabling forward cellular movement. **(D)** ECM degradation by enzymes can widen gaps through which the cell body can penetrate. AQP-1,−3,−4 and−9 are suggested to interact with ECM-degrading enzymes. **(E)** The final step is retraction of the cell trailing edge, thought to use aquaporins for water efflux following by K^+^ export.

Key molecular and cellular events involved in cell migration can be classified into five inter-dependent stages, which are polarization, protrusion, cell-matrix adhesion, extracellular matrix (ECM) degradation and retraction (Figure 2). Cell polarization provides functionally specialized domains in the membrane and cytoplasm (Drubin and Nelson, 1996), typified by asymmetric distributions of organelles, signaling mechanisms, and membrane channels, transporters and receptors (Swaney et al., 2010). In movement, changes in cell polarization generate leading and trailing edges, predominantly regulated by small GTPases such as CDC42 (Johnson and Pringle, 1990; Allen et al., 1998), which controls the recruitment of partitioning-defective (PAR) proteins, atypical protein kinase C (aPKC), and actin polymerization machinery (Etienne-Manneville and Hall, 2003; Goldstein and Macara, 2007). AQPs−1,−4,−5, and−9 have been shown to show polarized localization at the leading edges of migrating cells. Specific co-distributions with ion transporters such as the Na^+^/H^+^ exchanger, the Cl^−^/${\text{HCO}}_{3}^{-}$ exchanger, and the Na^+^/-HCO_3_ co-transporter, suggest sophisticated mechanisms for regulation of fluid influx and efflux (Loitto et al., 2002; Verkman, 2005; Hara-Chikuma and Verkman, 2006; Papadopoulos et al., 2008; Stroka et al., 2014), potentially driving membrane protrusions for cell locomotion (Schwab et al., 2007).

### Protrusion

A migrating cell extends its leading edge into the ECM by assembling a branched network of intracellular actin filaments, predicted to yield a physical force that dynamically pushes the membrane out, alternating with relaxation and actin depolymerization (Wang, 1985; Theriot and Mitchison, 1991; Pollard and Borisy, 2003). Membrane expansion requires the vesicle fusion to support the increase in surface area (Bretscher and Aguado-Velasco, 1998; Pierini et al., 2000; Fletcher and Rappoport, 2010). Three types of protrusions found in motile cells are lamellipodia, filopodia, and invadopodia. Lamellipodia are broad, flat, actin-rich protrusions that extend in the direction of locomotion and provide a foundation on which the cell moves forward (Cramer et al., 1997). Filopodia are long, thin protrusions of the membrane thought to be exploratory, “sensing” the local environment (Mattila and Lappalainen, 2008). Lamellipodial and filopodial formations are modulated by small GTPases in the Rho family, such as Rac1 and CDC42 (Ridley et al., 1992; Allen et al., 1997; Hall, 1998; Machesky, 2008), which stimulate actin polymerization in response to growth factor (Hall, 1998) and integrin receptor activations (Price et al., 1998). Interestingly, AQP9-facilitated water flux appears to critical for filopodial protrusion formation in fibroblasts, via the CDC42 pathway (Loitto et al., 2007). The Arp2/3 (actin-related protein 2/3) complex regulates the formation of new actin filaments in migrating cancer cells, and is regulated by Scar/WAVE complex (otherwise known as WANP), which interacts with the small GTPase Rac1 for lamellipodial assembly (Ibarra et al., 2005). Invadopodia are actin-rich, matrix-degrading protrusions that appear when ECM degradation and cell adhesion are needed to create space for movement, involving proteases such as MMP2, MMP9, and MT1-MMP and src tyrosine kinase (Weaver, 2006). Changes in cell volume during protrusion are assumed to require rapid water flow (Condeelis, 1993), and could occur in part in response to osmotic gradients governed by ion transport and actin polymerization state (Diez et al., 2005; Disanza et al., 2005; Schwab et al., 2007).

AQPs at the leading edges of migrating cells are well positioned to facilitate cell volume changes and cytoskeletal modifications during protrusion formation (Monzani et al., 2009; Jiang and Jiang, 2010; Klebe et al., 2015; Wei and Dong, 2015; Pelagalli et al., 2016). AQP1 overexpression in B16F10 melanoma cells and 4T1 mammary gland tumor cells enhanced cell migration and lamellipodial width *in vitro*, and augmented metastasis in a mouse model (Hu and Verkman, 2006). AQP1 is proposed to enhance lamellipodial formation by increasing membrane osmotic water permeability (Verkman, 2005; Hu and Verkman, 2006; Jiang, 2009), allowing water entry at the leading edge to impose hydrostatic pressure, drive membrane extension, and create space for actin polymerization. In addition to water channel activity, AQP1 is also thought to be an ion channel, proposed to allow gated conduction of monovalent cations through the central tetrameric pore (Anthony et al., 2000; Yu et al., 2006). The dual water and ion conductance of AQP1 is essential for colon cancer cell migration *in vitro* (Kourghi et al., 2015). Conversely, in clinical cases of cholangiocarcinoma, high AQP1 expression has been correlated with low metastasis (Aishima et al., 2007; Sekine et al., 2016), suggesting that AQP1 might play different roles in different types of cancers.

Other classes of AQP water channels are not necessarily interchangeable with AQP1 in facilitating cell migration (McCoy and Sontheimer, 2007), suggesting features of AQP1 other than simple osmotic water permeability are involved. AQP1-enhanced cell migration might also be due to interactions with cytoskeletal proteins. For example, Monzani et al. (2009) demonstrated that AQP1 knockdown dramatically impeded actin cytoskeletal organization in migrating human melanoma and endothelial cell lines via interaction with Lin-7/β-catenin. The Lin-7/β-catenin complex enables asymmetrical organization of filamentous actin (F-actin). AQP1 might act as a scaffolding protein at the leading edges. Jiang (2009) found that knocking down AQP1 was associated with re-localization of actin in migrating HT20 colon cancer cells, and a reduction in the activity of actin regulatory factors RhoA and Rac. A PDZ domain in Lin-7 could mediate interaction with rhotekin protein, which inhibits Rho GTPase signaling that is involved in cell migration, invasion, and cytoskeletal reorganization (Sudo et al., 2006). Rhotekin merits further evaluation in models of AQP1-dependent cytoskeletal organization.

A role for AQP4 in glioma cell migration has similarly been proposed to occur through regulation of cell volume and cytoskeletal interactions. Protein kinase C (PKC)-mediated phosphorylation of AQP4 at serine 180 correlated with a decreased glioma cell invasion (McCoy et al., 2010). AQP4-facilitated glioma invasion is dependent on co-expression of chloride channels (ClC2) and the potassium-chloride co-transporter 1 (KCC1) in invadopodia, which could provide the ionic driving force for water efflux leading to cell shrinkage that could augment invasiveness through ECM (Mcferrin and Sontheimer, 2006; McCoy et al., 2010). AQP4 effects on actin cytoskeleton suggest a role for α-syntrophin, interacting with the C-terminal domain of AQP4 at a PDZ-binding site (Neely et al., 2001). In human glioma and primary astrocytes, reduced AQP4 expression correlated with dramatic morphological elongation, reduced invasiveness, and impaired F-actin polymerization (Nicchia et al., 2005; Ding et al., 2011).

AQP5 facilitates protrusion formation, volume regulation, cell migration, and metastasis. AQP5 expression is correlated with cell invasiveness and metastasis of human prostate cancer (Li et al., 2014), lymph node metastasis in patients with colon cancer (Kang et al., 2015), and metastatic potential of lung cancer cells (Zhang et al., 2010). Moreover, Jung et al. (2011) showed that a shRNA-induced reduction in AQP5 expression in MCF7 breast cancer cells was associated with significantly reduced cell proliferation and migration. The mechanism of AQP5-facilitated cancer cell invasion and metastasis might be due to its direct or indirect interaction with the epidermal growth factor receptor/extracellular signal-regulated kinase (ERK1/2) pathway (Kang et al., 2008; Zhang et al., 2010), known to be important in cancer metastasis and aggressiveness (Vicent et al., 2004). Additionally, AQP5 mediates lung cancer cell membrane osmotic water permeability, and has been suggested to contribute to cancer cell migration and invasion by enabling rapid cell volume regulation and subsequent protrusion formation (Chen et al., 2011). The complementary role of ion transport for migration in AQP5-expressing cells was supported by Stroka et al. (2014), who found that cell migration through physically confined spaces occurred despite block of actin polymerization and myosin contraction, but relied on co-expression of the Na^+^/H^+^ exchanger with AQP5, supporting AQP5-induced cell volume regulation and its importance in cell motility.

AQP8 expression influences migration and invasion of cervical cancer cells, and AQP3 expression enhances pancreatic and colorectal cancer cell invasion and metastasis (Liu et al., 2012; Li et al., 2013; Shi et al., 2013). Further work is needed to investigate whether mechanisms of AQP3- and AQP8-facilitated cancer cell migration and invasion involve cell volume regulation, protrusion formation, cytoskeletal interaction, or other functional properties of the AQP channels that remain to be defined.

### Cell-matrix adhesion

Cell-matrix adhesions, first observed in cultured fibroblasts, connect the extracellular matrix to the actin cytoskeleton (Curtis, 1964). During migration, contacts with substratum must form to facilitate extension, and must detach to allow forward displacement of the cell. Insufficient anchoring causes protrusions to collapse, leading to a “membrane ruffling” phenomenon (Vicente-Manzanares and Horwitz, 2011). Protrusions adhere to ECM via integrin receptors, in turn linked to intracellular actin filaments (Ridley et al., 2003). The extracellular binding of integrin receptors to ECM ligands initiates integrin clustering, and activates protein tyrosine kinases and small GTPases. The organization of actin cytoskeleton and cell polarity controls the positions of focal adhesions for cell locomotion (Geiger et al., 2001; Martin et al., 2002). Cell-matrix adhesions create the focal points for generation of traction to pull the cell forward over the substratum.

Classes of aquaporins (AQP1-4) have been shown to interact with adhesion molecules and to influence adhesive properties of migrating cells. Increased AQP1 in mesenchymal stem cells enhances migration by a mechanism involving β-catenin and the focal adhesion kinase (FAK) (Meng et al., 2014), which regulates integrin signaling at focal adhesion sites (Schaller et al., 1992; McLean et al., 2005; Zhao and Guan, 2011). Whether AQP1 and FAK also interact in cancer cell migration remains to be tested. AQP2 appears to promote cell migration by modulating integrin β1 at focal adhesion sites, by a mechanism thought to involve an arginine-glycine-aspartate (RGD) motif in the second extracellular loop of AQP2 (Chen et al., 2012). When AQP2 is absent, integrin β1 is retained at focal adhesion sites, delaying recycling of focal adhesions, thus reducing migration rate. AQP2 also enables estradiol-induced migration and adhesion of endometrial carcinoma cells by mechanisms involving annexin-2 and reorganization of F-actin (Zou et al., 2011). Knockdown of AQP3 in human esophageal and oral squamous cell carcinoma with siRNA correlated with reduced phosphorylation of FAK, impaired cell adhesion and cell death (Kusayama et al., 2011); these effects would be predicted to impair cancer cell migration. AQP4 expression has been suggested to enhance cell-matrix adhesion in cancer cells (McCoy and Sontheimer, 2007). More research is needed to identify the intracellular signaling mechanisms and to determine whether other AQP classes alter cell migration via modulation of cell adhesion.

### ECM degradation

Extracellular matrix degradation widens pathways through which cells can penetrate tissues, and reduces the distortion of the rounded cell body needed for physical progress (Brinckerhoff and Matrisian, 2002; Mott and Werb, 2004). Invadopodia sprout from leading edge filopodia, extending through tiny channels in the ECM, and adhere to ECM collagen fibers (Weaver, 2006; Friedl and Wolf, 2009). To accommodate displacement of the cell body, constraining ECM fibers are cleared by local proteolysis, using surface proteases such as zinc-dependent matrix metalloproteinases (MMP) and serine proteases (Nagase and Woessner, 1999; Netzel-Arnett et al., 2003; Wolf et al., 2007). AQPs−1,−3,−4, and−9 have been shown to interact with specific MMPs to facilitate ECM degradation and invasion.

In lung cancer cells, migration was facilitated by AQP1 expression, linked to expression of MMP2 and MMP9 (Wei and Dong, 2015). In gastric cancer cells (SGC7901), AQP3 levels were correlated with MMP2, MMP9, and MT1-MMP levels, and enhanced invasiveness via phosphoinositide 3-kinase signaling (Xu et al., 2011). Positive correlations between AQP3, MMP2, and MMP9 and cancer invasiveness also occur in lung cancer (Xia et al., 2014; Xiong et al., 2017). In prostate cancer, AQP3 expression is correlated with up-regulation of MMP3 via ERK1/2 signaling, with increased cell motility and invasion (Chen et al., 2015). In glioma, AQP4 levels correlated with migration and invasiveness *in vitro* and *in vivo* through a mechanism involving MMP2 (Ding et al., 2011). AQP9 upregulation in prostate cancer could enhance growth, migration, and invasion involving ERK1/2 signaling; reduced levels of phosphorylated ERK1/2 and MMP9 were observed in AQP9-deficient cell lines (Chen et al., 2016). These studies suggest one of the key components of AQP-mediated facilitation of cancer cell invasion is the regulation of MMP proteases needed for degradation of ECM.

### Retraction

Following integrin-ligand binding, cross-linking proteins such as myosin II contract the actin filament strands (Vicente-Manzanares et al., 2009), developing tension against the intact adhesion points (Chrzanowska-Wodnicka and Burridge, 1996). The final step in the cycle of cell movement is retraction of the trailing edge. A working model is that membrane tension opens stretch-activated Ca^2+^ channels, activating calpain and triggering disassembly of focal adhesion proteins on the trailing edge, while concurrent K^+^ efflux drives volume loss at the cell rear, resulting in detachment and net translocation along the substrate. In this model, the role of AQP channels is to facilitate osmotic water efflux in response to K^+^ efflux (Huttenlocher et al., 1997; Palecek et al., 1998; Schwab et al., 2007) presumably in parallel with electroneutral efflux of chloride ions.

## AQP pharmacology and therapeutic implications in cancer invasion and metastasis

Aquaporin pharmacological agents have attracted keen interest for their potential therapeutic uses in diseases involving impaired fluid homeostasis. Aquaporins in cancer metastasis are new translational targets for AQP modulators. Known and proposed inhibitors of AQPs include cysteine-reactive metals such as mercury (II) chloride (HgCl_2_) (Preston et al., 1993), gold-based compounds (Martins et al., 2013), carbonic anhydrase inhibitor acetazolamide (Ma et al., 2004a; Gao et al., 2006), and small molecule inhibitors such as tetraethylammonium (TEA^+^) (Brooks et al., 2000), although the small molecule blockers vary in efficacy between preparations. The pharmacological panel for AQPs has been expanding steadily, with new compounds being discovered around the world, including for example the University of Niigata, Japan (Huber et al., 2009), Radboud University, Netherlands (Detmers et al., 2006), the Faculty of Pharmacy, University of Lisbon, Portugal (Martins et al., 2012), the Institute of Food and Agricultural Research and Technology, Barcelona, Spain (Seeliger et al., 2012), the University of Adelaide, Australia (Niemietz and Tyerman, 2002; Yool, 2007), the University of Groningen, Netherlands (Martins et al., 2013), the University of Kiel, Germany (Wu et al., 2008), and others. This review focuses specifically on selected AQP pharmacological agents that to date have been tested in models of cancer cell migration and metastasis (Table 2).

**Table 2:** Summary of AQP pharmacology used in cancer invasion and metastasis.

**Molecule name**	**Molecular structure**	**AQP activity**	**Effect**
TEA^+^	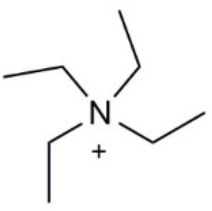	•Inhibits AQP1, AQP2, and AQP4 water flux (Brooks et al., 2000; Yool et al., 2002; Detmers et al., 2006)	•Inhibits osteosarcoma and hepatocellular carcinoma cell migration and invasion (*in vitro*) (Pelagalli et al., 2016)
Acetazolamide	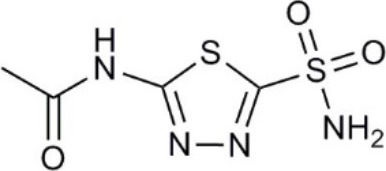	•Inhibits AQP1 and AQP4 water flux (Ma et al., 2004a; Tanimura et al., 2009) •Suppresses AQP1 expression (Xiang et al., 2004)	•Inhibits angiogenesis and metastasis in Lewis lung carcinoma (*in vivo*) (Xiang et al., 2002, 2004) •Suppresses tumor growth in colon cancer (*in vivo*) (Bin and Shi-Peng, 2011)
Topiramate	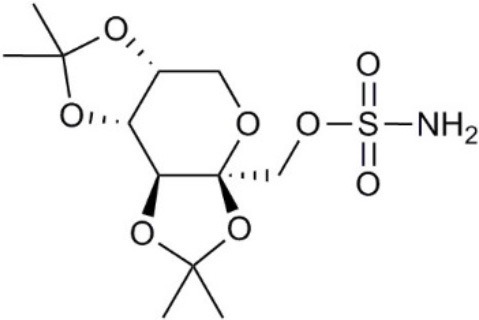	•Suppresses AQP1 expression (Ma et al., 2004b)	•Suppresses Lewis lung carcinoma growth and metastasis (*in vivo*) (Ma et al., 2004b)
AqB007	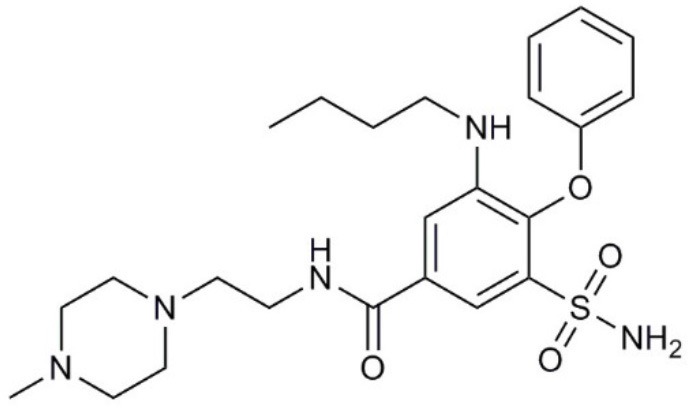	•Inhibits AQP1 ion flux (Kourghi et al., 2015)	•Inhibits colon cancer cell migration (*in vitro*) (Kourghi et al., 2015)
AqB011	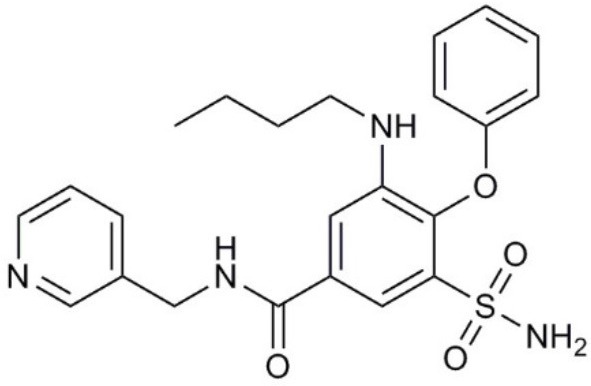	•Inhibits AQP1 ion flux (Kourghi et al., 2015)	•Inhibits colon cancer cell migration (*in vitro*) (Kourghi et al., 2015)
AqB013	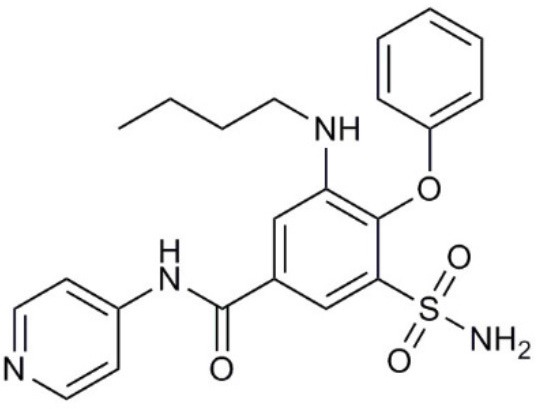	•Inhibits AQP1 and AQP4 water flux (Migliati et al., 2009)	•Inhibits endothelial tube formation and colon cancer cell migration (*in vitro*) (Dorward et al., 2016)
Bacopaside I	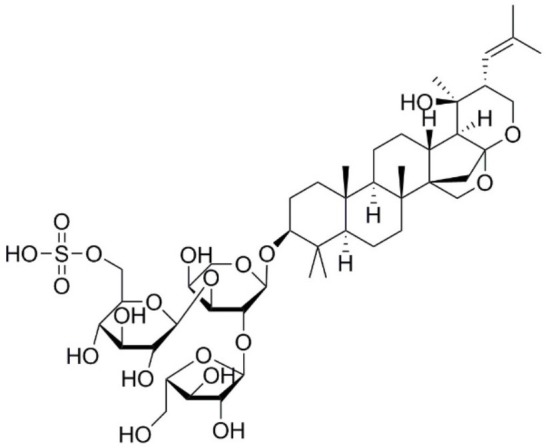	•Inhibits AQP1 water flux (Pei et al., 2016)	•Inhibits colon cancer cell migration (*in vitro*) (Pei et al., 2016)
Curcumin	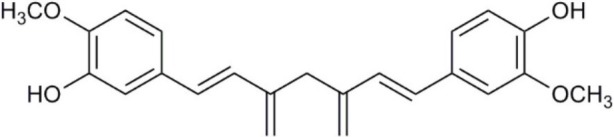	•Inhibits EGF-induced AQP3 upregulation (Ji et al., 2008)	•Inhibits ovarian cancer cell migration (*in vitro*) (Ji et al., 2008)
Bacopaside II	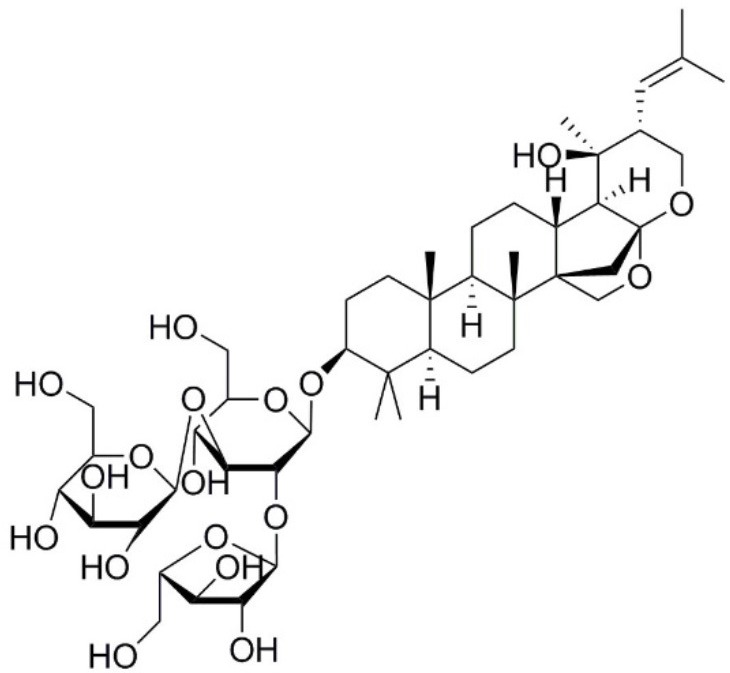	•Inhibits AQP1 water flux (Pei et al., 2016)	•Inhibits colon cancer cell migration (*in vitro*) (Pei et al., 2016)
Ginsenoside Rg3	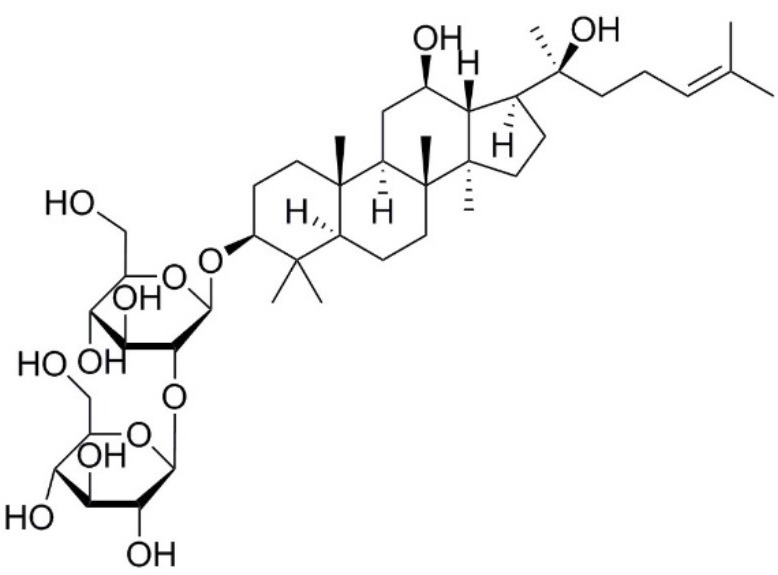	•Suppresses AQP1 expression (Pan et al., 2012)	•Inhibits prostate cancer cell migration (*in vitro*) (Pan et al., 2012)

### Acetazolamide and topiramate

Acetazolamide and topiramate are FDA-approved drugs that inhibit carbonic anhydrase. Acetazolamide at 100 μM was reported to inhibit water channel activity by 39% for AQP1 expressed in human embryonic kidney (HEK293) cells (Gao et al., 2006), and by 81% at 10 μM in the *Xenopus* oocyte expression system (Ma et al., 2004a). AQP4 activity was inhibited by 47% at 1,250 μM in proteoliposomes (Tanimura et al., 2009). However, acetazolamide (at doses up to 10,000 μM) did not block water flux in erythrocytes with native AQP1 expression, or epithelial cells transfected with AQP1 (Yang et al., 2006; Søgaard and Zeuthen, 2008). Acetazolamide inhibited angiogenesis in a chick chorioallantoic membrane assay, and tumor growth and metastasis in mice with Lewis lung carcinoma (Xiang et al., 2002, 2004), perhaps as a result of reduced AQP1 expression (Bin and Shi-Peng, 2011). Topiramate reduces Lewis lung carcinoma growth and metastasis, with effects similarly attributed to suppression of AQP1 expression (Ma et al., 2004b). It will be of interest to compare the effects of acetazolamide and topiramate on angiogenesis, tumor growth, and metastasis with those of AQP1 channel inhibitors.

### Tetraethylammonium

TEA^+^ is an inhibitor of voltage-gated potassium channels, calcium-dependent potassium channels, the nicotinic acetylcholine receptor, and it has also been shown to block AQP-1,−2, and−4 water permeability in *Xenopus laevis* oocytes and kidney derived cell lines (Brooks et al., 2000; Yool et al., 2002; Detmers et al., 2006). However, inhibition of AQP1 water permeability by TEA^+^ is variable, having been confirmed by some groups (Detmers et al., 2006), and challenged by others (Søgaard and Zeuthen, 2008). Yang et al. (2006) reported no block of water flux by TEA^+^ in erythrocytes with native AQP1, or in epithelial cells transfected with AQP1, and suggested previous positive results might have been due to inhibition of K^+^ channels and altered baseline cell volume; however, the observation that site-directed mutation of AQP1 altered TEA sensitivity (Brooks et al., 2000) ruled out this alternative explanation. TEA^+^ block of AQP1 water permeability reduced cell migration and invasion in *in vitro* models of osteosarcoma and hepatocellular carcinoma (Pelagalli et al., 2016), with outcomes interpreted as consistent with action of TEA^+^ as a possible AQP1 inhibitor. However, given the variability in efficacy and cross-talk with other channels, TEA^+^ is not an ideal candidate for clinical development, although the targets causing the observed block of cancer cell migration and invasion might merit further investigation.

### Bumetanide derivatives

Bumetanide is a sulfamoylanthranilic acid derivative used clinically to increase diuresis by blocking sodium cotransporter activity at the loop of Henle in the nephron. Molecular derivatives of bumetanide have been synthesized and found to exhibit inhibitory effects on classes of AQP channels. For example, the bumetanide derivative AqB013 blocks osmotic water fluxes mediated by mammalian AQP1 and AQP4 channels expressed in *Xenopus laevis* oocytes (Migliati et al., 2009). The water channel blocker AqB013 was shown to inhibit endothelial tube formation and colon cancer cell migration and invasion *in vitro* (Dorward et al., 2016). Other bumetanide derivatives, AqB011 and AqB007, block the AQP1 ion conductance, but not water flux (Kourghi et al., 2015). In AQP1, the central tetrameric pore is thought to be permeable to monovalent cations, CO_2_, and NO (Nakhoul et al., 1998; Herrera et al., 2006; Yu et al., 2006; Musa-Aziz et al., 2009), although some work questioned AQP1-mediated CO_2_ and cation transport properties (Yang et al., 2000; Fang et al., 2002; Tsunoda et al., 2004). An ionic conductance in AQP1-expressing *Xenopus* oocytes stimulated with forskolin was first reported in 1996 (Yool et al., 1996); however, the forskolin response proved to be inconsistent when repeated by other groups (Agre et al., 1997). Further work showed the forskolin effect was indirect; the direct regulation of the AQP1 cation conductance depended on cGMP binding (Anthony et al., 2000). The reason that AQP1 cation channels have low opening probability (Saparov et al., 2001) or are not detectable (Tsunoda et al., 2004) reflects the availability of AQP1 to be gated by cGMP, which depends on tyrosine phosphorylation status of the carboxyl terminal domain, suggesting the AQP1 ion channel function is highly regulated (Campbell et al., 2012). With the discovery of AQP1 ion blocking agents, AqB011 and AqB007, the physiological function of the ion channel activity could finally be addressed. When applied to AQP1-expressing HT29 colon cancer cells, these inhibitory compounds significantly reduced cancer cell motility (Kourghi et al., 2015), suggesting a physiological role of AQP1 ion conductance in cell migration. Mutation of the candidate binding site in the AQP1 intracellular loop D domain removed sensitivity to AqB011, showing that the inhibitory mechanism directly involved the AQP1 channel and could not readily be attributed to off-target actions on other channels or transporters (Kourghi et al., 2018). Another bumetanide derivative AqB050 was shown to inhibit mesothelioma cell motility and metastatic potential *in vitro*, but not *in vivo* (Klebe et al., 2015). The mechanism of action of AqB050 in blocking mesothelioma cell motility *in vitro* remains to be determined.

### Plant-based derivatives

Plant-based derivatives that reduce cancer cell migration and invasion include agents that have also have been found to inhibit AQPs. Bacopa monnieri is a perennial herb native to the wetlands of India that is used in alternative medicinal therapies. Chemical constituents bacopaside-I and bacopaside-II, were shown to block AQP1 but not AQP4 water channels (Pei et al., 2016). Pei and colleagues also found that bacopaside-I and bacopaside-II attenuated migration of colon cancer cell lines expressing high levels of AQP1, but had no effect on lines with low AQP1, suggesting the inhibitory effects were AQP1-specific. Ginsenoside Rg3 from a traditional Asian medicinal plant Panax ginseng is an intriguing candidate for possible anti-metastatic therapies. Ginsenoside Rg3 inhibited prostate cancer cell migration and was associated with downregulation of AQP1 expression via the p38 MAPK pathway and transcription factors (Pan et al., 2012). Effects of Ginsenoside Rg3 directly on water channel activity, or on expression levels of other aquaporins, remain unknown. Curcumin is a naturally occurring ingredient in turmeric, used as therapeutic tool for pathologies including cancer (Gupta et al., 2013). Curcumin was found to inhibit EGF-induced upregulation of AQP3 and migration in human ovarian cancer cells, via inhibition of AKT/ERK and PI3K pathways (Ji et al., 2008); however, curcumin affects a number of biochemical pathways and might not be suited when AQP-specific modulation is required (Aggarwal et al., 2003). Research on the effects of curcumin in other cancers such as gastric cancer, in which EGF-induced AQP3 up-regulation occurs, might further understanding of the role of AQP3 in cell migration and invasion (Huang et al., 2010).

### Metal-based inhibitors

Mercury has classically been used as an AQP1 inhibitor. In the human AQP1 monomer, the NPA motif in loop E is near cysteine 189, which is the site at which mercury inhibits osmotic water permeability (Preston et al., 1993). Lack of a cysteine in the corresponding position is consistent with mercury insensitivity in mammalian AQP4 (Preston et al., 1993). However, mercury is not a promising candidate for AQP-specific modulation or therapeutic application due to its toxicity and non-specific side-effects. Metal-based inhibitors that have been tested in models of cancer include AQP3 inhibitors such as NiCl_2_ (Zelenina et al., 2003) and CuSO_4_ (Zelenina et al., 2004), which inhibited EGF-induced cell migration in human ovarian cancer cells. Auphen is a gold-based compound which, when administered at concentrations of 100 μM, blocks AQP3 glycerol transport by 90%, and water transport by 20% in human red blood cells (Martins et al., 2012). Auphen also blocks proliferation in various mammalian cell lines, including human epidermoid carcinoma, by inhibiting AQP3 glycerol transport (Serna et al., 2014). This merits more research into the importance of AQP3-facilitated glycerol transport in cancer invasiveness, and whether gold-based compounds such as auphen can also be used to suppress cancer invasion and metastasis.

## Conclusion

Aquaporin-dependent mechanisms serve as key steps throughout the process of metastasis, in angiogenesis, cellular dissociation, cell migration and invasion. AQPs−1,−2,−3,−4,−5,−8, and−9 contribute to one or more processes, generally potentiating cancer invasion and metastasis by boosting tumor angiogenesis, enhancing cell volume regulation, regulating cell-cell and cell-matrix adhesions, interacting with the actin cytoskeleton, regulating proteases and ECM degrading molecules, contributing to the regulation of epithelial-mesenchymal transition in cancer cells, and interacting with specific signaling pathways important in cancer cell motility and invasions. Pharmacological agents for aquaporin channels have therapeutic promise for improving cancer treatment, and include derivatives of bumetanide, organic metal compounds, plant medicinal agents, and other small molecule compounds. Although conflicting evidence has been raised for some compounds, there is nevertheless a compelling need to continue identifying novel candidates for AQP-specific modulators relevant not only for the treatment of cancer, but other pathological conditions. In conclusion, although much remains to be defined for molecular mechanisms in cancer invasion and metastasis, the roles of AQP channel function in cancer progression will inspire new therapeutic targets for improving treatment of malignant and invasive carcinomas.

## Author contributions

MD: wrote the manuscript; AY: reviewed and edited the manuscript.

### Conflict of interest statement

The authors declare that the research was conducted in the absence of any commercial or financial relationships that could be construed as a potential conflict of interest.
